# The Synthesis of Backbone Thermo and pH Responsive Hyperbranched Poly(Bis(*N*,*N*-Propyl Acryl Amide))s by RAFT

**DOI:** 10.3390/polym8040135

**Published:** 2016-04-08

**Authors:** Shijiao Zhou, Dongxin Zhang, Libin Bai, Jing Zhao, Yonggang Wu, Hongchi Zhao, Xinwu Ba

**Affiliations:** 1 College of Chemistry and Environmental Science, Hebei University, Baoding 071002, China; zhoushijiao@126.com (S.Z.); dongxin0225@126.com (D.Z.); wuyonggang@hbu.edu.cn (Y.W.); zhc@hbu.edu.cn (H.Z.); 2 Center of Comprehensive Experimental, Hebei University, Baoding 071002, China; liufengzz27@163.com

**Keywords:** hyperbranched, backbone, thermo-pH response, RAFT, poly(bis(*N*,*N*-propyl acrylamide))

## Abstract

Hyperbranched poly(methylene-bis-acrylamide), poly(bis(*N*,*N*-propyl acryl amide)) (HPNPAM) and poly(bis(*N*,*N*-butyl acryl amide)) were synthesized by reversible addition-fragmentation chain transfer polymerization. HPNPAMs showed lower critical solution temperature (LCST) due to an appropriate ratio between hydrophilic and hydrophobic groups. The effects of reaction conditions on polymerization were investigated in detail. The structure of HPNPAM was characterized by ^1^H NMR, FT-IR, Muti detector-size exclusion chromatography (MDSEC) and Ultravioletvisble (UV-Vis). The α value reached 0.20 and DB was 90%, indicating HPNPAMs with compact topology structure were successfully prepared. LCSTs were tuned by *M*_w_ and the pH value of the solution. The change of molecular size was assayed by dynamic light scattering and scanning electron microscope. These results indicated that the stable uniform nanomicelles were destroyed and macromolecules aggregated together, forming large particles as temperature exceeded LCST. In addition, after the cells were incubated for 24 h, the cell viability reached 80%, which confirmed this new dual responsive HPNPAM had low cytotoxicity.

## 1. Introduction

Thermo-pH dual-responsive polymers have attracted interest in the stimuli-responsive materials field for their specific properties of temperature-pH response. Among the past research, there have been a large number of thermo-pH dual-responsive polymers reported, most of which are linear polymers, such as poly(*N*-isopropylacrylamide), poly(*N*,*N*-diethylacrylamide), poly-(methylvinylethe), poly(*N*-vinylcaprolactam), poly(2-(dimethylamino)ethylmethacrylate) [1]. With the investigation of the hyperbranched polymer, because of the combination of the advantages of stimulus response and the unique properties of hyperbranched structure, such as high solubility, low viscosity, and the abundance of terminal groups, thermo-pH dual-responsive hyperbranched polymers have attracted increasing interest, and are promising for applications in terms of physical separation [2], biomedical [3,4,5], and other fields [6]. Currently, temperature-pH dual-responsive hyperbranched polymers can be divided into two categories. One is terminal temperature-pH dual responsive polymers, that is, the temperature and pH sensitive groups or segments are grafted onto the hyperbranched polymer surface, which is the most widely researched [7,8,9,10,11,12,13]. However, the other, called backbone temperature-pH dual responsive hyperbranched polymers, is almost stagnant. In recent years, Yan et al have made groundbreaking progress in the backbone temperature-sensitive hyperbranched polymers and have prepared a variety of backbone temperature-sensitive hyperbranched polymers [14,15,16,17,18]. Because of the limitations of epoxy monomers, only proton transfer or anionic polymerization could be selected to synthesize backbone temperature response hyperbranched polyethers. Therefore, it is important to find other monomers that can be adopted to more polymeric methods, such as free radical polymerization and living control polymerization.

The pioneering work of Sherrington made important progress in the preparation of highly branched macromolecules by controlled crosslinked of divinyl monomers [19]. In our previous work, bis(*N*,*N*-ethyl acrylamide) was designed and used to synthesize a hyperbranched polymer via reversible addition-fragmentation chain transfer polymerization (RAFT). Fortunately, the as-prepared hyperbranched poly(bis(*N*,*N*-ethyl acryl amide)) showed temperature and pH response properties due to the similar structure with NIPAM. Because the monomer has two double bonds, the whole monomer was blocked into the molecular chain, which resulted in the temperature and pH response coming from the molecular backbone [20]. Surprisingly, the HPNPAM could be prepared by free radical, ionic and living radical polymerization under mild reaction conditions due to the high reactivity of acryl group.

Although the hyperbranched poly(bis(*N*,*N*-ethyl acryl amide)) showed the backbone temperature and pH response, whether this means that polymers with similar structure can exhibit the backbone temperature and pH response is a key question to confirm its value. Herein, methylene-bis-acrylamide, bis(*N*,*N*-propyl acryl amide) and bis(*N*,*N*-butyl acryl amide) were selected as the monomers. The corresponding hyperbranched polymers were synthesized to investigate the backbone temperature and pH response. The structures of the hyperbranched polymers were characterized by nuclear magnetic resonance spectroscopy (NMR) and Fourier transform infrared spectroscopy (FT-IR). The molecular weight and molecular weight distribution of the polymers were determined by muti detector-size exclusion chromatography (MD-SEC), and the lower critical solution temperature (LCST) was studied by UV-Visible spectrum. In addition, the effect of reaction conditions on the conversion rate, molecular weight, molecular weight distribution and the degree of branching was investigated in detail.

## 2. Experimental Section

### 2.1. Materials

Methylene-bis-acrylamide(MAM), *N*,*N*-dimethylformamide(DMF), ammonium persulfate (APS) and acetone (95%, Tianjin kemiou chemical reagent Co., Tianjin, China) needed to be purified before use. The bis(*N*,*N*-propyl acryl amide) (NPAM), bis(*N*,*N*-butyl acryl amide) (NBAM) and *S*,*S*′-bis(a,a-dimethyl-a′′-acetic acid)-trithiocarbonate (BDAAT) were synthesized following a literature procedure [21]. Distilled deionized water was prepared from a Millipore Filtration System (Millipore, MA, USA). Concentrated hydrochloric acid was used (Sinopharm Chemical Reagent Co., Ltd, Shanghai, China).

### 2.2. Methods

Infrared spectroscopy was measured on a Varian 640-IR Fourier infrared spectrometer (Varian, Palo Alto, CA, USA) by using KBr pellets. ^1^H NMR spectra were recorded on a Bruker AVANCE-600 600 MHz spectrometer (Bruker, Germany). The chemical shifts are given in parts per million (ppm). Light-transmittance of the polymer solution was measured on a temperature-controlled UV-Visible 2550 Spectrophotometer (Shimadzu, Japan) at 500 nm with the heating rate as 0.1 °C·min^−1^. LCSTs were defined as the temperature corresponding to 50% transmittance of an aqueous solution during the heating process, and the curves of transmittance *versus* temperature of all samples are inserted in supplementary files (Figures S4–S6). Molecular weight, polydispersity, Mark-Houwink index (α) and intrinsic viscosity (IV) were determined by a Viscotek 270-MDSEC (Malvern Instruments Ltd., Malvern, PA, USA) equipped with the differential refractive index (RI), viscometer, and two-angle light scattering (LS, Malvern Instruments Ltd., Malvern, PA, USA) triplet detectors. The eluent was 0.1 mol·L^−1^ NaNO_3_ aqueous solution at a flow rate of 1 mL·min^−1^ at 15 °C. For MDSEC, narrow dispersion polyethylene oxide std-PE022K was used to calibrate the instrument. The morphology of the polymer aggregation was characterized by a JSM-7500 Scanning electron microscope (SEM, JEOL Ltd., Tokyo, Japan). As a representative example for sample preparation, a drop of solution (L4) was added on the slide that was frozen quickly by liquid nitrogen to keep the true morphology, then the sample with solid membrane was obtained by lyophilization. After gold powder was sprayed onto the surface of sample, SEM was used to study the morphology. Dynamic Light Scattering (DLS) measurements were performed on a Brookhaven BI-200 goniometer (Brookhaven, New York, NY, USA) with vertically polarized incident light of wavelength λ = 532 nm supplied by a heliumneon laser operated at 75 mW and a Brookhaven BI-4700 AT digital autocorrelator (Brookhaven, New York, NY, USA). Polymer cytotoxicity was detected by MTT assay. The photocytotoxicity of HPNPAM on HeLa cells was examined as follows: HeLa cells (5 × 10^3^ cells) in 100 μL of DMEM containing 10% FCS were plated in a 96-well plate and incubated for 24 h in a humidified atmosphere of 5% CO_2_ in air at 37 °C (Sanyo, Model MCO-18AIC, Osaka, Japan) Then, 100 μL of an HPNPAM in DMEM containing 10% FCS and 2% DMSO was added to each well. Incubation was carried out for 6 (12, 24 h) at an HPNPAM concentration of 0.5 μM (final DMSO content was 1% in all cases). Cells without HPNPAM treatment were used as the control group. After 6 (12, 24 h) of treatment, MTT dye solution (20 μL, 5 mg·mL^−1^) was added to each cell. Cells were incubated for another 4 h and then analyzed using a microplate spectrophotometer (BioRad Model 3550, CA, USA) at 570 nm. The percentage cell survival was calculated by normalization with respect to the value without HPNPAM treatment.

### 2.3. Synthesis of Hyperbranched Poly(bis(N,N-Propyl Acryl Amide) (HPNPAM)

Taking the hyperbranched poly(bis(*N*,*N*-propyl acryl amide) as an example, the monomer, BDAAT, APS and purified DMF were added to a Schlenk tube. Oxygen was removed by repeated vacuum-nitrogen cycles. Then, the polymerization was conducted at 70 °C in an oil bath for a desired reaction time. Afterwards, the obtained polymers were precipitated by dropping the solution into a large excess of acetone to remove the remaining small molecules. The precipitated polymers were separated by centrifugation, redissolved in a minimum amount of water, and reprecipitated into acetone. Finally, the products were freeze-dried from water and weighed to obtain the conversion. Conversion of the resulting polymer was calculated by weighing in accordance with the following formula:

$$Conversion\%=\frac{polymer}{monomer+CTA+I}\times 100\%$$

## 3. Results and Discussion

### 3.1. The Reaction Mechanism of HPNPAM

**Scheme 1:**
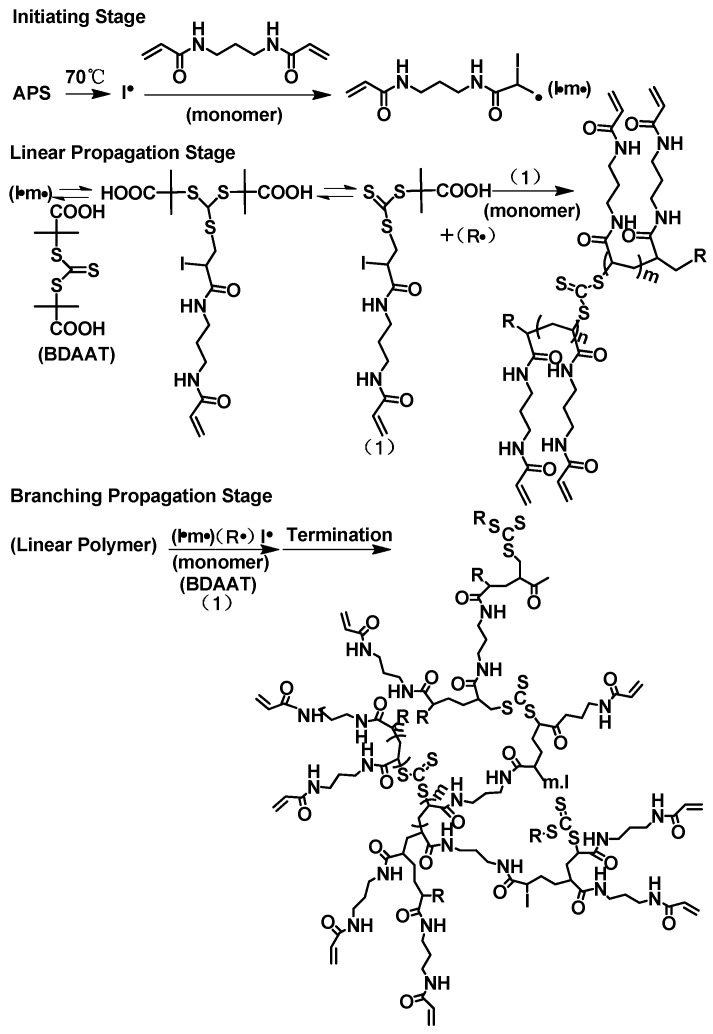
The reaction mechanism of HPNPAM. (1): dormant species

Taking the HPNPAM as an example, the reaction mechanism of HPNPAM is outlined in Scheme 1. In the initiating stage, the initiator APS is decomposed to initial free radical under 70 °C, then the monomers are initiated by the initial free radicals that are consistent with the established mechanism of RAFT polymerization [21]. In the branching propagation stage, the vinyl groups as the side groups possess reaction activity and can be initiated by the active species (I^•^, R^•^, and I-m^•^). Therefore, the branched structure or degree of branching (DB) will be increased with the conversion of the vinyl groups. For the multi vinyl monomer, it will be polymerized, forming a crosslinked polymer rather than a hyperbranched polymer due to the high reactivity of vinyl groups. Thus, RAFT has to be occupied for slowing down the growth rate and avoiding rapid cross-linking. Finally, the HPNPAMs are obtained bearing amount of vinyl groups and thioester.

### 3.2. The Parameters of HPMAM, HPNPAM and HPNBAM

Hyperbranched poly(methylene-bis-acrylamide) (HPMAM), poly(bis(*N*,*N*-propyl acryl amide)) and poly(bis(*N*,*N*-butyl acryl amide)) (HPNBAM) were prepared by RAFT. The data are listed in Table 1. The parameters of the polymers are similar. The conversion reached around 50%, the polydispersity index (PID) shows the characteristics of wide dispersion, and the topology structure exhibits the feature of hyperbranched, which is proved by the DB and Mark-Houwink index (α). However, the phenomenon we expected that L-a, L-b and L-c would show a lower critical solution temperature (LCST) did not appear. L-a is a hydrophilic polymer, L-b is a hydrophobic polymer, only L-c is an amphiphilic polymer and shows the LCST. For these three kinds of monomers, the number of carbons is the key reason. As the number of carbons increases, the hydrophobic nature of the monomer is stronger and the feature of the hyperbranched polymer is changed from hydrophilic to amphiphilic, then to hydrophobic.

### 3.3. Characterization for HPNPAMs

#### 3.3.1. The Degree of Branching for HPNPAMs

**Figure 1:**
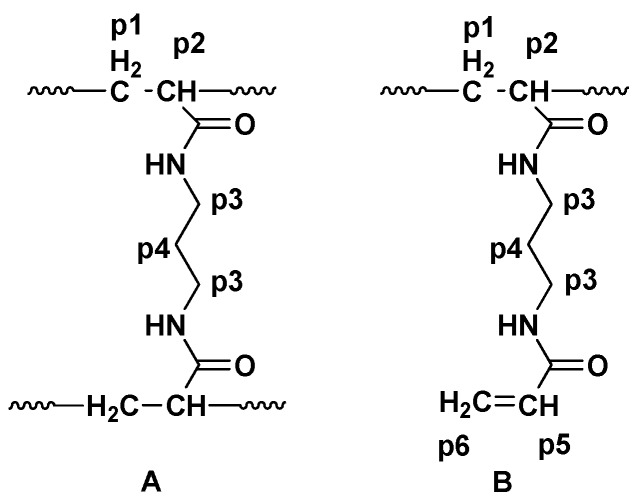
A = dendritic units, B = linear unit, terminal unit.

**Figure 2:**
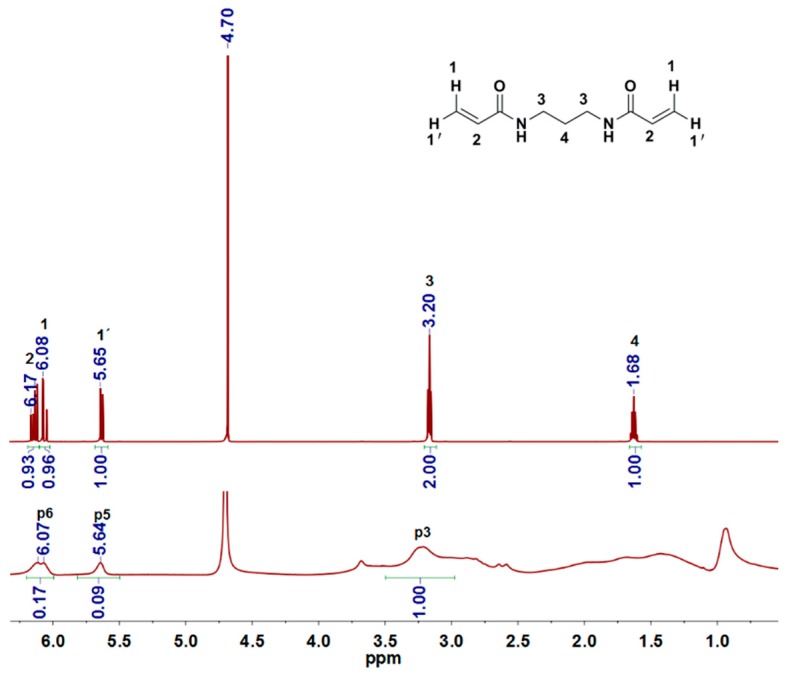
^1^H NMR spectra of hyperbranched polymer and monomer.

The structure of HPNPAMs was characterized by IR spectra, ^1^H NMR spectra and MDSEC. The results of ^1^H NMR spectra and MDSEC revealed the topology structure information including DB and α. Frey pointed out that DB was defined as the ratio between the number of dendritic units and the total number of units. Thus, DB can be calculated according to the integration ratio of different protons by using ^1^H NMR. For HPNPAMs, there are three kinds of units as shown in Figure 1. A is the dendritic unit, B is the linear unit or terminal unit. The different protons are labeled as p1, p2, p3, p4, p5 and p6. Both dendritic units and linear units or terminal units include protons of p3 and p4, therefore the total number of units can be represented by the area of p3 or p4. However, the chemical shift of p4 is overlapped with p1, thus the area of p3 is used to represent the total number of units in this paper. Assuming that there is no side reaction, the molecular chain is made up of linear units, therefore the integration ratio of p3 and p6 should be 2:1. However, the pendant vinyl groups are initiated to form branched chains, leading to the decrease in area of p6. Obviously, the decrement of the area of p6 is attributed to the dendritic units, which can be calculated by the equation (p3 − 2 × p6). Therefore, the equation for DB is written as DB = (p3 − 2 × p6)/p3. ^1^H NMR for sample 7 is displayed in Figure 2 (^1^H NMR spectras for the rest samples are shown in Figure S3). The areas of p6 and p3 are shown under the corresponding peaks. The DB of sample 7 is 0.66 according to the aforementioned equation.

#### 3.3.2. α Value of HPNPAMs

**Figure 3:**
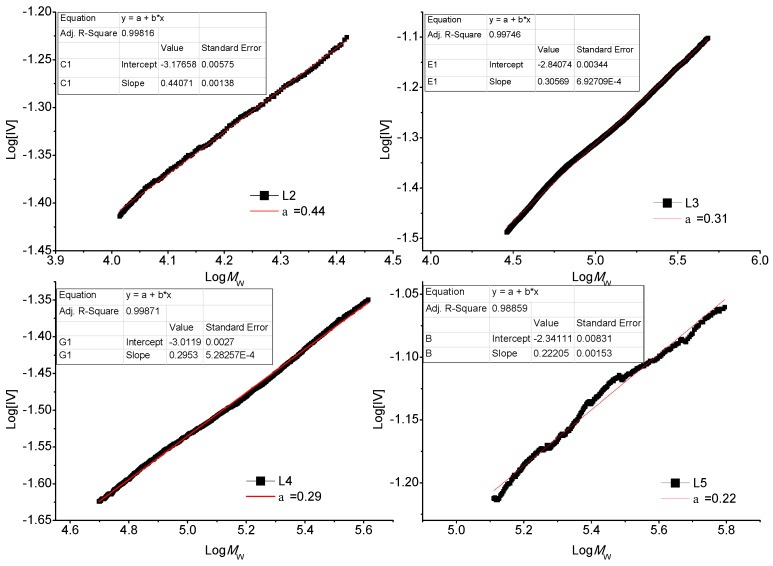
The plots of Log*M*_w_ *vs.* Logŋ.

**Figure 4:**
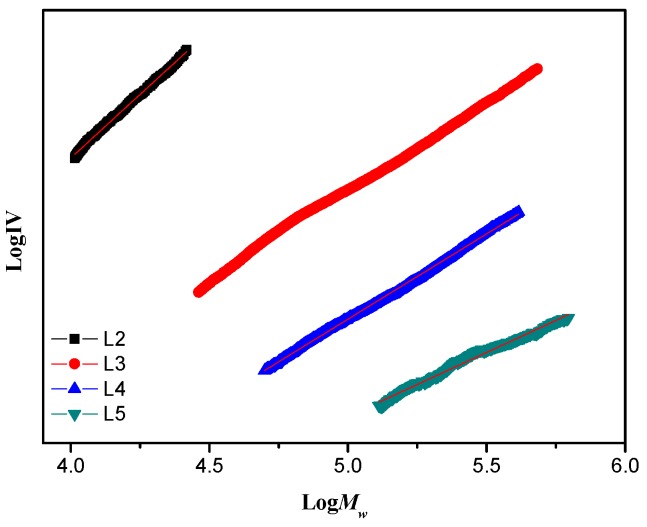
The plots of Log*M*_w_ *vs*. Logŋ (L2 α = 0.44, L3 α = 0.31, L4 α = 0.29, L5 α = 0.22).

Molecular weight of HPNPAMs were determined by a Viscotek 270-MDSEC equipped with RI, viscometer, and LS triplet detectors. Since this instrument is able to measure *M*_w_ and intrinsic viscosity (ŋ) at the same time, the software can show α by Log*M*_w_ *vs.* Logŋ. Although α cannot give the branch number for hyperbranched polymers, the trend of α can reflect information about branching for hyperbranched polymers under the same testing condition. The plots of Log*M*_w_ *vs*. Logŋ for sample L2, L3, L4 and L5 are shown in the Figure 3. After the plot was linearly fitted, the α value was shown as 0.44, 0.31, 0.29 and 0.22, respectively. Usually, the α value of linear polyacrylamide is 0.75 in the water system. The α values of the hyperbranched polymers are lower than 0.5, which suggest a more compact hyperbranched structure of the products. By combining these plots in one figure, we can obtain more information. As shown in Figure 4 (other plots are shown in Figure S2), the slope of these lines show a decreasing trend. Furthermore, from Figure 4, it is easy to observe that Logŋ decreases in turn when the Log*M*_w_ has the same value, which further proves that DB increases with reaction temperature.

#### 3.3.3. FTIR Analysis of Monomer and Polymer

**Figure 5:**
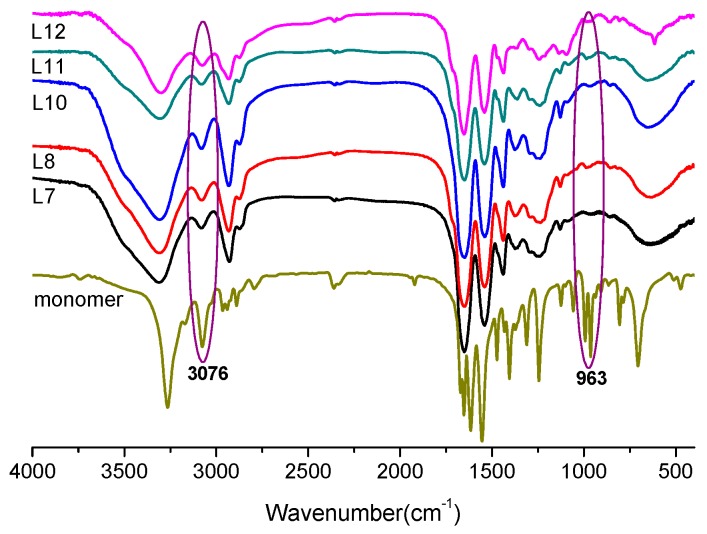
FTIR spectra of monomer and hyperbranched polymers.

IR spectra were also occupied to characterize the structure of HPNPAMs and are shown in Figure 5 and Figure S1. Based on the reaction mechanism, part of vinyl groups would disappear after polymerization. The characteristic absorption peaks of =C–H are located at 963 and 3076 cm^−1^ in the IR spectra. As expected, compared with monomer, the intensity of peaks located at 963 and 3076 cm^−1^ obviously become weak, which indicates the number of vinyl groups that took part in the polymerization. The peaks of 963 and 3076 cm^−1^ are assigned to pendant vinyl originating from the linear units and terminal units of HPNPAMs.

### 3.4. Effect of Reaction Conditions on Polymerization

#### 3.4.1. Effect of Reaction Temperature on Polymerization

A series of HPNPAMs were prepared to investigate the effect of reaction conditions on the polymerization. Temperature was selected as one of the factors. The obtained data are shown in Table 2. When the temperature was set as 40 °C, lower than the initiator decomposition temperature, polymerization was not conducted. When the temperature was higher than the initiator decomposition temperature, the number of primary radicals in the reaction system gradually increased with temperature, which resulted in much higher *M*_w_ and conversion. In addition, the DB of HPNPAMs also increased. The activity of the chain transfer agent (CTA) is the key reason. For the RAFT polymerization, the reversible equilibrium constant is higher as the temperature increases. One one hand, it is helpful to control the polymerization and avoid the formation of hydrogel. On the other hand, numerous vinyl groups took part in polymerization, forming a short chain under the initiation of primary radicals or CTA, which brought a higher DB to HPNPAM. The Mark-Houwink index (α) of HPNPAMs also reflects the same trend of DB when the α values were gradually decreased over the temperature range of 60–90 °C. The LCSTs decreased from 15 to 9 °C as the reaction temperature increased. This was attributed to *M*_w_ or DB. When the reaction temperature reached 120 °C, thermo-polymerization could not be circumvented and broke the reversible equilibrium of RAFT, leading to the formation of hydrogel.

#### 3.4.2. Effect of Reaction Time on Polymerization

**Figure 6:**
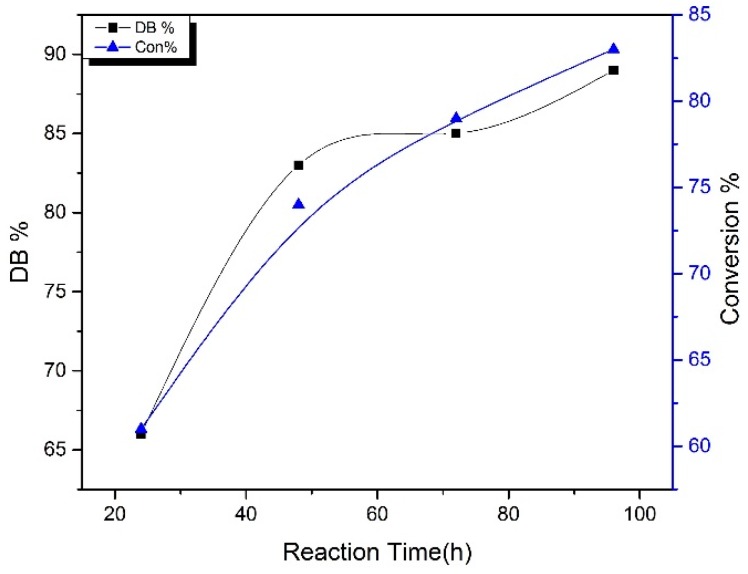
The reaction time *vs.* DB% and the reaction time *vs*. Conversion%.

Reaction time, an important factor for RAFT, was studied in detail and shown in Table 3. The trends of *M*_w_ and Conversion are consistent with the rule of RAFT. With an extension in the reaction time, much more monomers were initiated by I^•^, R^•^, and I-m^•^, therefore the Conversion, *M*_w_ and PID showed an increasing trend. The trend of DB and α reflected that DB was increased by extending the reaction time. To graphically display the trend of DB, the data of DB *vs* Reaction time are shown in Figure 6. DB increases sharply in the first 48 h and then shows a slow increase in the subsequent 48 h. Compared with the Conversion, the reason is that the increment of Conversion is decreased during the period 48–96 h, which means the reaction rate (R_p_) is lower. On the one hand, a long reaction time increases the number of dormant-breaking dormant process, thus more monomers are captured by the polymer chains, and with the consumption of monomer, the reaction rate decreases, and gel formation is effectively avoided. On the other hand, the propagation of the main chain and side chain increase slowly, resulting in the increment of *M*_w_ slowing down. The reaction probability of vinyl coming from hyperbranched polymers is also decreased, which is not conducive to forming much more branch chains, thus the DB shows a slow increase.

#### 3.4.3. Effect of CTA’s Concentration on Polymerization

Table 4 is the effect of CTA’s concentration on polymerization. According to the rule of RAFT, the activity of free radicals R^•^ can initiate the polymerization; however, the intermediate radicals as the dormant radicals in the system cannot initiate monomers. Therefore, the polymerization rate will decrease when the CTA’s concentration increases. As the concentration of CTA reached 5 mmol, no polymers were collected by using the method of precipitation, which could be attributed to the lower polymerization rate that resulted from the chain transfer reaction at high CTA concentration. On the contrary, DB displays an increase trend compared with M_w_ and Conversion. This is consistent with our previous work. In the reaction system, the propagating chain free radicals possess large steric hindrance; it is much easier to initiate the vinyl of monomers. Compared with propagating chain free radicals, steric hindrance of initial free radicals is small, which mitigates the steric hindrance effect and improves the reaction probability of pendant vinyl, leading to higher DB. As the RAFT reaction mechanism points out, the number of initial free radicals will increase along with the increase in the CTA’s concentration, which is helpful to improve DB for HPNPAMs.

### 3.5. Temperature Response Performance of HPNPAMs

**Figure 7:**
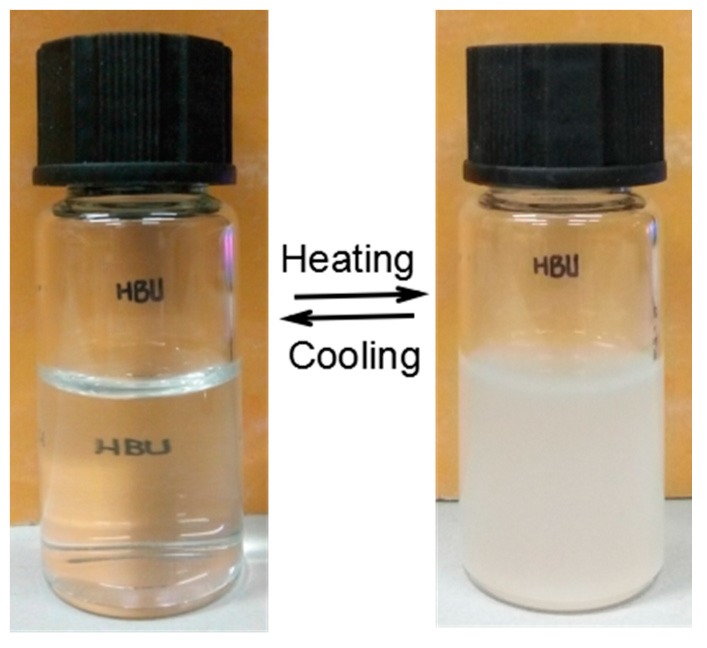
Photographs of an aqueous solution of L2. 5 °C (**left**) and 25 °C (**right**); HBU: Hebei University.

**Figure 8:**
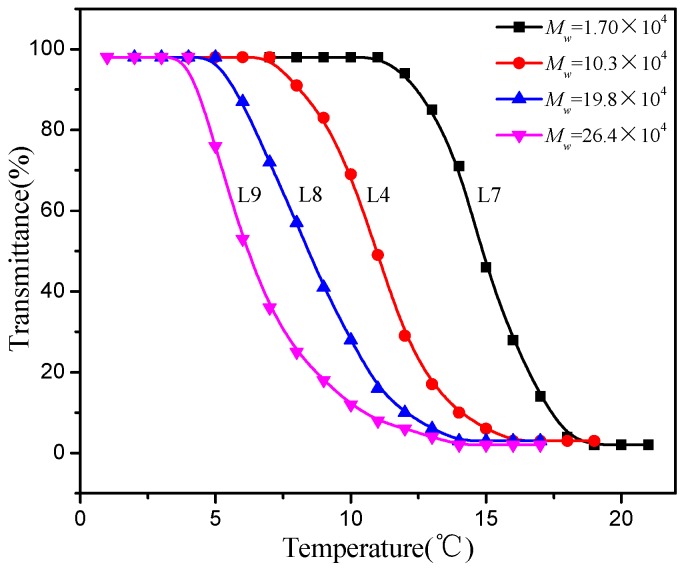
The curves of transmittance *versus* temperature of L4, L7, L8 and L9. Solution concentration: 3 mg·mL^−1^; heating rate: 0.1 °C·min^−1^; pH = 7.

As shown in Figure 7, at a relatively low temperature, a certain concentration of the polymer solution is transparent. As the temperature increases, the solution gradually becomes cloudy, and this change process is reversible. This phenomenon indicates that the HPNPAM has a temperature sensitive performance. For the kind of poly(acrylamide)s, the phenomenon of LCST is concerned with the number of carbons or the ratios of hydrophilic and hydrophobic groups as proved by the results of Table 1. Screening out the data with the same molecular weight and different DB as standard, these data are listed in Table 5. Their LCST values are closed although there is a big gap between the DB, which indicates that DB has little influence on LCST. If DB is not the major reason for the different LCST values, molecular weights will be the key factor. As Zhou pointed out, the macromolecules with higher *M*_w_ will undergo the phase transition first [22]. LCST curves of L4, L7, L8 and L9 are shown in Figure 8. LCST increased with molecular weights, which is consistent with Zhou’s conclusion. The same trend also appears in Table 2 and Table 4.

### 3.6. pH Response Performance of HPNPAMs

**Figure 9:**
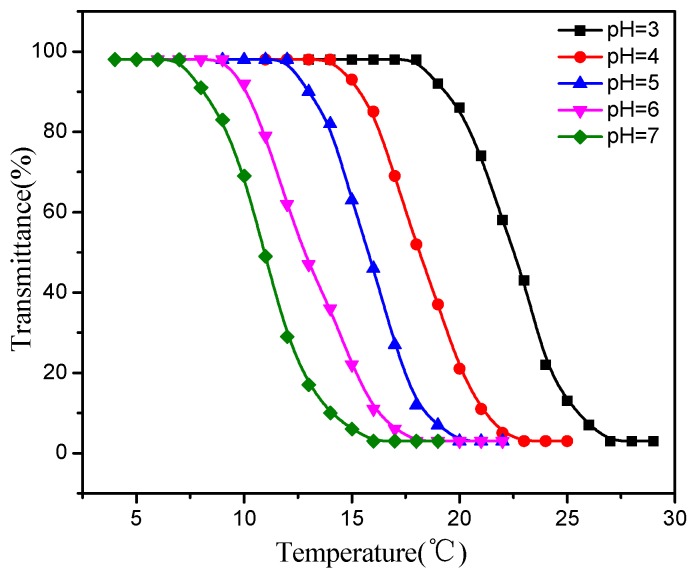
The curves of transmittance *versus* temperature of HPNPAMs. Aqueous solution with different pH values. Solution concentration: 3 mg·mL^−1^; heating rate: 0.1 °C·min^−1^.

For HPNPAMs, amide groups can combine with acid, leading to the change of the dissolved condition. Therefore, LCST should be tuned by the pH of the solution. The results are shown in Figure 9. LCST is tuned from 11 to 23 °C when the pH value decreases from 7 to 3. The acidity of the solution is stronger, the number of protonated amide groups is higher, which provide stronger hydrophilic property, resulting in LCST gradually increasing.

### 3.7. DLS and SEM Analysis

**Figure 10:**
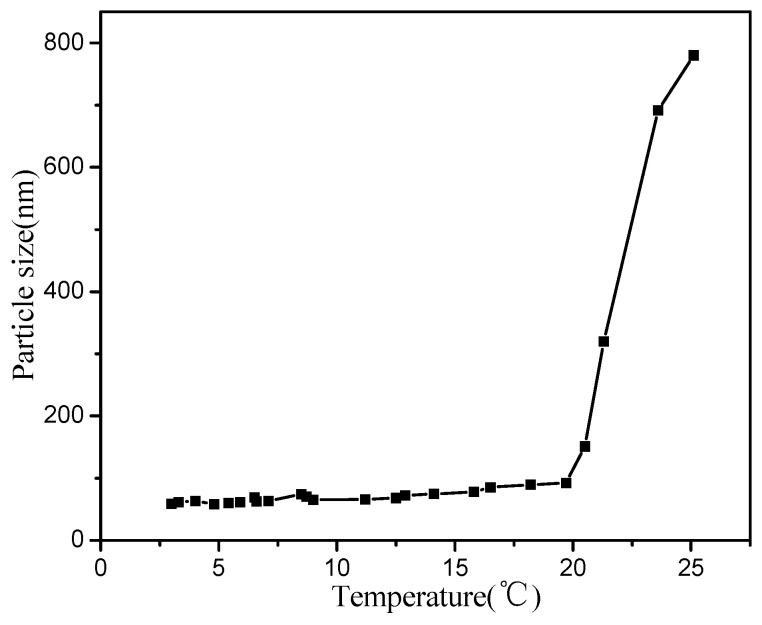
The diameter of nanomicell *vs.* Temperature for each temperature, measuring the diameter after holding the temperature for 10 min. Solution concentration: 2 mg·mL^−1^; heating rate: 0.1 aqueous·min^−1^; pH = 4.

**Figure 11:**
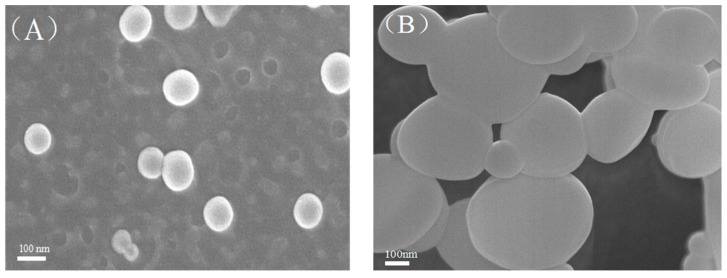
The SEM of polymer solutions with different temperatures ((**A)** < LCST, (**B)** > LCST).

DLS and SEM were further employed to investigate the mechanism for LCST. As previously reported, nanomicells aggregate to large particles as the temperature is above the cloud point, which results in the decrease of transmittance for the polymers’ solution. Because backbone-thermoresponsive hyperbranched polymers are composed of hydrophilic and hydrophobic segments in the backbone, uniform nanomicelles can be formed when the concentration of the polymers’ solution is high. As shown in Figure 10, the particle size of L4 in aqueous solution (pH = 4) was kept around 80 nm when the temperature was below the LCST, which indicates that HPNPAM molecules form uniform nanomicelles. This conclusion was also supported by the SEM. As described in the Methods section, a drop of solution (L4) was quickly frozen by liquid nitrogen to keep the true morphology. In Figure 11A, globular particles with sizes of 60–100 nm were dispersed on the slide surface.

Therefore, for a sample with *M*_w_ (1.04 × 10^5^), a reasonable explanation is HPNPAM molecules form uniform nanomicelles due to the amphiphilic macromolecules backbone. When the temperature was higher than the LCST, hydrogen bonds between HPNPAM and the solvent were broken. HPNPAM only showed a hydrophobic property; thus, the stable uniform nanomicelles were destroyed and macromolecules aggregated together forming large particles. Figure 10 and Figure 11B display the evidence to support this mechanism. The size could reach 800 nm when the temperature increased to 25 °C. Compared with the scale length, the particle size was 300–400 nm or even greater when the temperature was above the LCST.

### 3.8. Cytotoxicity Test of HPNPAM

**Figure 12:**
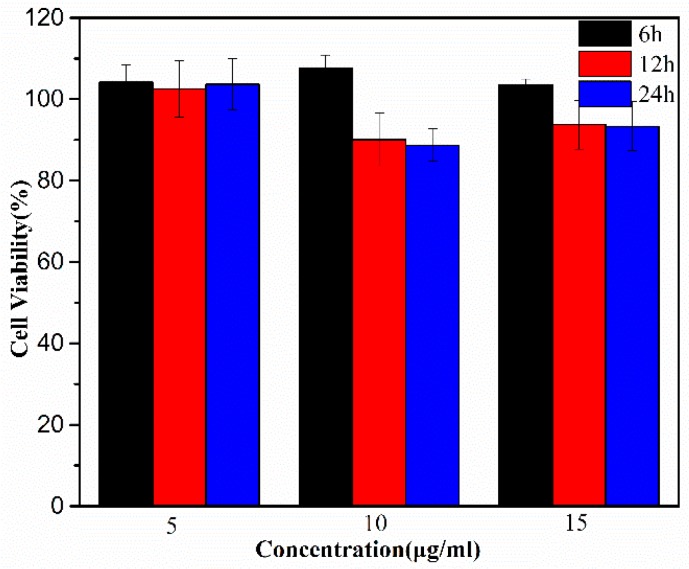
Cell viability of the obtained polymer at different concentrations.

In the biomedical field, cell viability is often used to determine the cytotoxicity and biocompatibility for materials. The cytotoxicity against Hela cells was studied by using the MTT assay. The resulting HPNPAM is homopolymer, the composition of every sample is the same, only with different *M*_w_ and DB, so it is reasonable that only a sample for the cytotoxicity assays represents all the samples. Figure 12 shows the cell viability after incubation with L12 at various concentrations. The cell viability was around 100% for all of the concentrations after 6 h incubation with L12. The cell viability could reach 80% even when the cells were incubated for 24 h with concentration of 15 μg·mL^−1^. The cytotoxicity test results showed that the toxicity of HPNPAM in a short period of time was very low. As everyone knows, the half-life of the drug determines its carrier’s residence time in the cell, and the half-life of the drug is usually less than 24 h; after 24 h, the carrier material may also be discharged through metabolism. Therefore, the HPNPAM’s low cytotoxicity has initially led to its potential applications for drug delivery systems.

## 4. Conclusions

In conclusion, hyperbranched poly(bis(*N*,*N*-propyl acryl amide)) was obtained by using RAFT polymerization as a method. The correctness of HPNPAM was confirmed by IR and NMR spectroscopy. We discussed in detail the effects of reaction conditions on the polymer molecular weight, degree of branching and monomer conversion. The results demonstrated that within a certain range of temperature and time, molecular weight, degree of branching of the polymer and monomer conversion gradually increase as the temperature rises. In addition, more chain transfer agents can make the polymer molecular weight and monomer conversion rates relatively low, as well as narrow the molecular weight distribution and increase the degree of branching. In addition, the thermo-sensitivity was also discussed in this paper, and the result showed that the HPNPAM has temperature-sensitive responsiveness and pH responsiveness. Cytotoxicity tests show that the HPNPAM has low cytotoxicity and exhibits potential for biomedical applications.

## Figures and Tables

**Table 1:** The parameters of HPMAM, HPNPAM and HPNBAM.

Samples	Monomer:CTA:I (mmol)	Temperature	*M*_w_ (× 10^4^)	*M*_w_/*M*_n_	Conversion	α	DB	LCST
L-a	20:1:1	70 °C	5.6	2.14	60%	0.43	70%	-
L-b	20:1:1	70 °C	3.5	2.08	48%	-	60%	-
L-c	20:1:1	70 °C	4.7	2.21	53%	0.32	74%	13 °C

L-a: methylene-bis-acrylamide, Time = 0.3 h, DMF = 6 mL; L-b: bis(*N*,*N*-butyl acryl amide), Time = 48 h, DMF = 6 mL, I = BPO, *M*_w_ = 3.5 × 10^4^, *M*_w_ was determined by Shodex GPC using 0.01 mol·L^−1^ LiBr/DMF solution at a flow rate of 1 mL·min^−1^ at 15 °C. A series of narrow dispersion polystyrene was used to calibrate a standard curve; L-c: bis(*N*,*N*-propyl acryl amide), Time = 48 h, DMF = 4 mL.

**Table 2:** Effect of reaction temperature on polymerization.

Samples	Temperature	*M*_w_ (× 10^4^)	*M*_w_/*M*_n_	Conversion	α	DB	LCST
L1	40 °C	–	–	–	–	–	–
L2	50 °C	1.4	2.52	41%	0.44	55%	15 °C
L3	70 °C	4.7	2.21	53%	0.31	74%	13 °C
L4	80 °C	10.3	1.97	74%	0.29	83%	11 °C
L5	90 °C	25.7	2.72	89%	0.22	88%	9 °C
L6	120 °C	gel	–	–	–	–	–

Monomer:CTA:I = 20:1:1 mmol; Time: 48 h; DMF = 4 mL.

**Table 3:** Effect of reaction time on polymerization.

Samples	Time	*M*_w_ (× 10^4^)	*M*_w_/*M*_n_	Conversion	α	DB	LCST
L7	24 h	1.7	1.85	61%	0.33	66%	15 °C
L4	48 h	10.3	1.97	74%	0.29	83%	11 °C
L8	72 h	19.8	2.43	79%	0.27	85%	8 °C
L9	96 h	26,4	2.98	83%	0.22	89%	6 °C

Monomer:CTA:I = 20:1:1 mmol; Temperature: 80 °C; DMF = 4 mL.

**Table 4:** Effect of CTA’s concentration on polymerization.

Samples	Monomer:CTA:I (mmol)	*M*_w_ (× 10^4^)	*M*_w_/*M*_n_	Conversion	α	DB	LCST
L10	20:0.5:1	14.4	2.68	86%	0.33	72%	7 °C
L4	20:1:1	10,3	1.97	74%	0.29	83%	11 °C
L11	20:2:1	6.9	1.71	69%	0.27	86%	14 °C
L12	20:3:1	1.3	1.54	43%	0.20	90%	17 °C
L13	20:4:1	–	–	–	–	–	–

Temperature: 80 °C, Time = 24 h, DMF = 4 mL.

**Table 5:** Effect of DB% on LCST of HPNPAMs.

Samples	*M*_w_ (× 10^4^)	*M*_w_/*M*_n_	Conversion	α	DB	LCST
L7	1.7	1.85	61%	0.43	66%	15 °C
L2	1.4	2.52	41%	0.44	55%	15 °C
L12	1.3	1.54	43%	0.20	90%	17 °C

## Supplementary Materials

The following are available online at www.mdpi.com/2073-4360/8/4/135/s1. Figure S1: FTIR spectra of monomer and hyperbranched polymers; Figure S2: The plots of Log*M*_w_ *vs.* Logŋ (L10 α = 0.44, L4 α = 0.29, L11 α = 0.276, L12 α = 0.20); Figure S3: ^1^H NMR spectra of L2, L3, L4, L5, L7, L8, L9, L10, L11, L12; Figure S4: The curves of transmittance *versus* temperature of L2, L3, L4 and L5 (Reaction temperature: L2 = 50 °C, L3 = 70 °C, L4 = 80 °C, L5 = 90 °C); Figure S5: The curves of transmittance *versus* temperature of L7, L4, L8 and L9 (Reaction time: L7 = 24 h, L4 = 48 h, L8 = 72 h, L9 = 96 h); Figure S6: The curves of transmittance *versus* temperature of L10, L4, L11 and L12 ((Monomer:CTA:I): L10 = (20:0.5:1), L4 = (20:1:1), L11 = (20:1.5:1), L12 = (20:2:1)).
