# Supplementary material for: The Synthesis of Backbone Thermo and pH Responsive Hyperbranched Poly(Bis(N,N-Propyl Acryl Amide))s by RAFT
Source: Polymers (Basel). 2016 Apr 8;8(4):135. doi: 10.3390/polym8040135 (PMC6432253; doi:10.3390/polym8040135)
Supplement: Supplementary file 1 [file polymers-08-00135-s001.pdf]

Supplementary Materials: The Synthesis of Backbone Thermo and pH Responsive Hyperbranched Poly(Bis(*N*,*N*-Propyl Acryl Amide))s by RAFT

Shijiao Zhou, Dongxin Zhang, Libin Bai, Jing Zhao, Yonggang Wu, Hongchi Zhao and Xinwu Ba

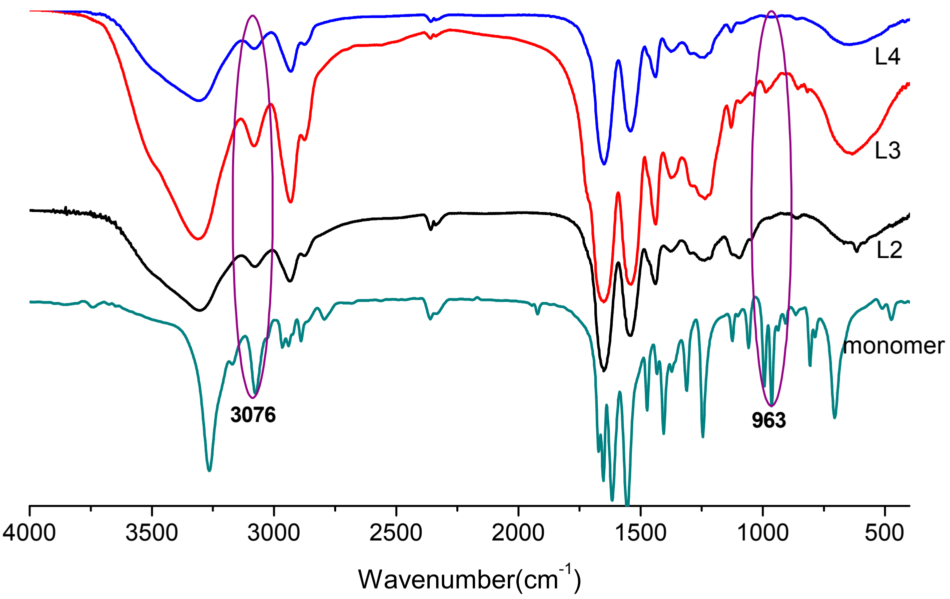

Figure S1. FTIR spectra of monomer and hyperbranched polymers.

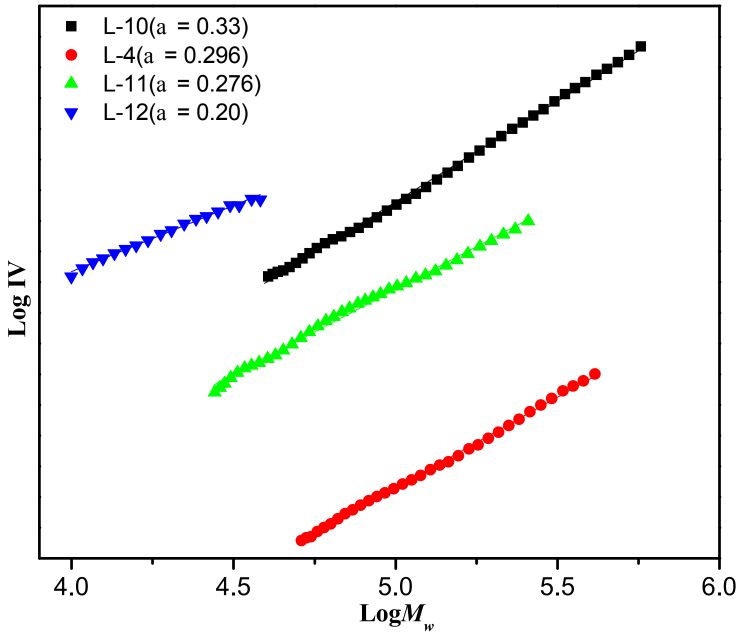

Figure S2. The plots of LogM_w *vs.* Logη (L10 $\alpha = 0.44$, L4 $\alpha = 0.29$, L11 $\alpha = 0.276$, L12 $\alpha = 0.20$).

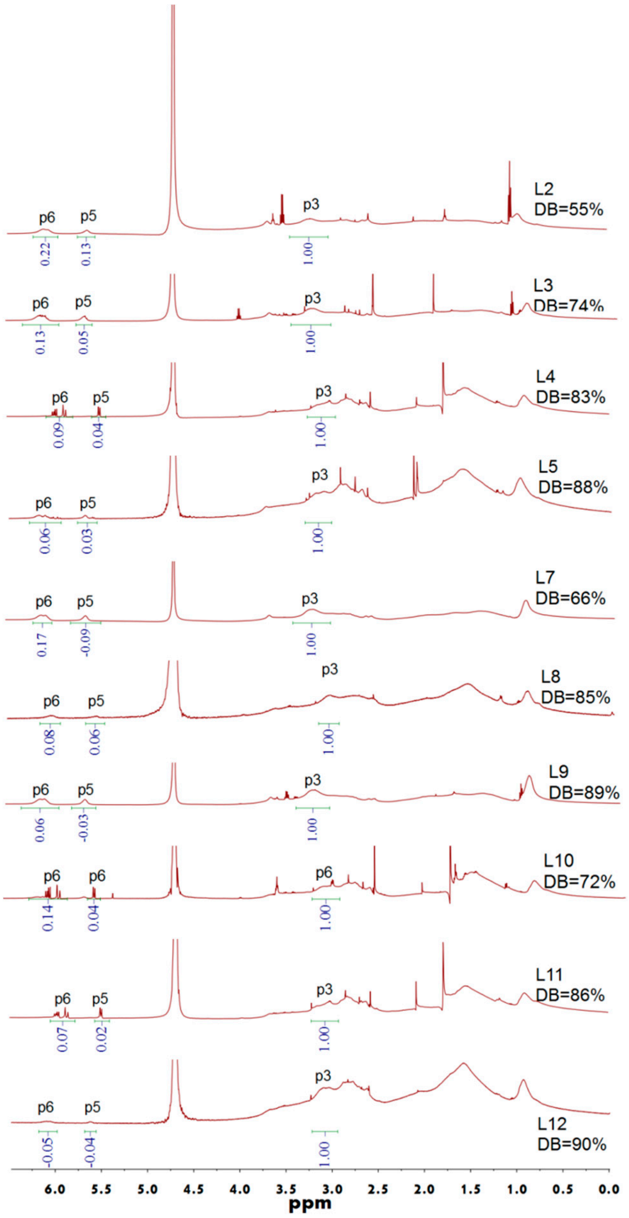

Figure S3. ^{1}H NMR spectra of L2, L3, L4, L5, L7, L8, L9, L10, L11, L12.

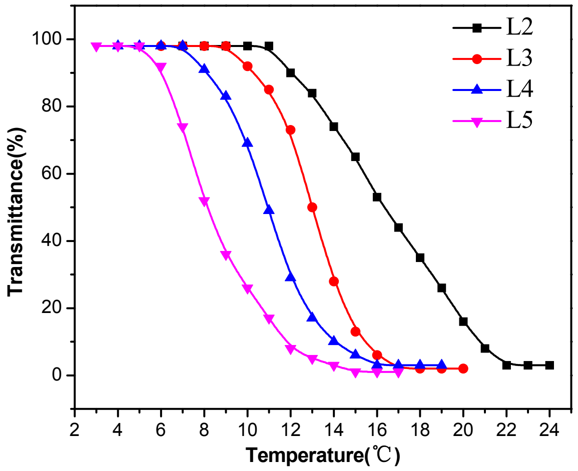

Figure S4. The curves of transmittance versus temperature of L2, L3, L4 and L5. Reaction temperature: L2 = 50 °C, L3 = 70 °C, L4 = 80 °C, L5 = 90 °C.

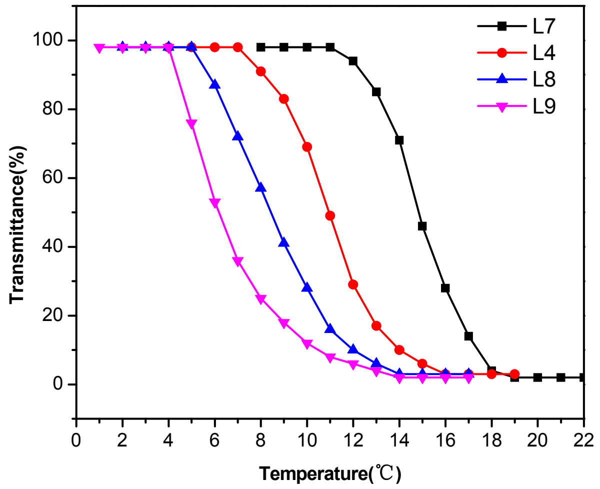

Figure S5. The curves of transmittance versus temperature of L7, L4, L8 and L9. Reaction time: L7 = 24 h, L4 = 48 h, L8 = 72 h, L9 = 96 h.

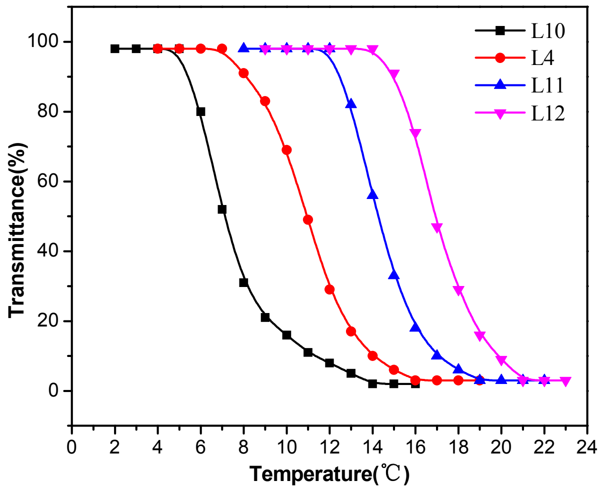

Figure S6. The curves of transmittance *versus* temperature of L10, L4, L11 and L12. (Monomer:CTA:I):L10 = (20:0.5:1), L4 = (20:1:1), L11 = (20:1.5:1), L12 = (20:2:1).

© 2016 by the authors; licensee MDPI, Basel, Switzerland. This article is an open access article distributed under the terms and conditions of the Creative Commons by Attribution (CC-BY) license (http://creativecommons.org/licenses/by/4.0/).
